# Application of “mosiac sign” on T2-WI in predicting the consistency of pituitary neuroendocrine tumors

**DOI:** 10.3389/fsurg.2022.922626

**Published:** 2022-07-26

**Authors:** Ding Nie, Peng Zhao, Chuzhong Li, Chunhui Liu, Haibo Zhu, Songbai Gui, Yazhuo Zhang, Lei Cao

**Affiliations:** ^1^ Beijing Neurosurgical Institute, Capital Medical University, Beijing, China; ^2^ Department of Neurosurgery, Beijing Tiantan Hospital, Capital Medical University, Beijing, China

**Keywords:** T2-weighted imaging, mosaic sign, consistency, surgical approaches, magnetic resonance imaging, PitNETs

## Abstract

**Purpose:**

Tumor consistency is important for pituitary neuroendocrine tumors (PitNETs) resection to improve surgical outcomes. In this study, we evaluated the T2-WI of PitNETs and defined a specific T2-WI signaling manifestation, the “Mosaic sign,” to predict tumor consistency and resection of PitNETs.

**Design:**

A retrospective review of MRI and tumor histology of 137 consecutive patients who underwent endoscopic endonasal resection for PitNETs was performed.

**Methods:**

The “Mosaic sign” was defined by the ratio of the tumor itself T2-WI signals, and characterized by multiple intratumor hyperintense dots. The degree of tumor resection was an assessment by postoperative MRI examination. The presence of the “Mosaic sign” was compared with patients' basic information, tumor consistency, tumor pathological staining, and surgical result. To determine whether the presence or absence of “Mosaic sign” could predict tumor consistency and guide surgical resection of tumors.

**Results:**

Statistical analysis showed that the consistency of the tumor and the degree of resection were correlated with the “Mosaic sign”. In the 137 cases of T2-WI, 43 had “Mosaic sign”, 39 cases had soft tumor consistency, and 4 were classified as fibrous, of which 42 were completely resected and 1 was subtotal resected. Of the 94 patients without “Mosaic sign”, the consistency of tumor of 54 cases were classified as soft, the remaining 40 cases were fibrous, 80 cases were completely resected, and 14 cases were subtotal resected. Postoperative cerebrospinal fluid leakage occurred in 1 patient. The number of corticotroph adenomas in the group of “Mosaic sign” was higher, with the statistical difference between the two groups (*P* = 0.0343).

**Conclusions:**

The presence of the “Mosaic sign” in T2-WI may provide preoperative information for pituitary adenomas consistency and effectively guide surgical approaches.

## Introduction

PitNET is a kind of common benign intracranial tumor (1). The transsphenoidal approach has been the preferred treatment for the vast majority of PitNETs. However, for 1%–4% of these tumors, a transcranial approach is still required (2). The choice of surgical approach remains a problem for tumors with extensive suprasellar and lateral extension (3). However, if tumor consistency can be predicted preoperatively, the choice of surgical approach in the face of these complex types of adenomas may no longer bother the surgeon (4–6). When the tumor is soft, it can be fully removed by aspiration and curettage *via* the transsphenoidal approach. However, when the tumor is fibrous, it is difficult to be completely resected. Sometimes, craniotomy is even necessary to achieve a satisfactory resected effect (4, 7). Magnetic resonance imaging (MRI) is an important means of preoperative examination for PitNETs, which can provide information including tumor location, size, and aggressiveness (8, 9). The predictive value of MRI in PitNET consistency is being continuously explored (10). Conventional MRI methods such as T1-WI and T2-WI as well as CE-FIESTA and DWI have been shown to predict tumor consistency in PitNETs (11–14). However, the reliability of the forecasts is controversial (15). The purpose of our study was to determine whether preoperative MRI features might be associated with tumor consistency. Specifically, we analyzed the relationship between the “Mosaic sign” in T2-WI and tumor consistency and surgical outcome to test whether it could effectively predict tumor consistency to provide guidelines for surgeons in planning operations.

## Materials and methods

### Patients

This retrospective study included all patients with histopathologically proven PitNETs who underwent transsphenoidal resection of tumors at our hospital between January 2020 and February 2021, and who underwent MRI before and after surgery. A total of 137 patients met the inclusion criteria. All patients were followed up until now. The study was approved by our hospital's Institutional Review Board.

### Clinical setting

All patients were operated on by the same team of neurosurgeons. Tumor consistency, classified as soft or fibrous, was assessed in blinded double-check by the two surgeons according to the lesions' inner surgical features after reviewing their surgical notes and video records. In detail, tumors easily removable with conventional maneuvers of curettage and suction were defined as soft. More resistant ones, difficult to remove and thus requiring more complex maneuvers such as extracapsular dissection, were classified as fibrous (11). Although this definition is quite subjective, it is widely used and does make sense to surgeons (16).

### Imaging

The scans were performed using a 3.0-Tesla MRI scanner (Magnetom Avanto). All patients underwent MRI before and after surgery, and T2-WI (TR 4000 ms, TE 89 ms, layer thickness 4 mm, layer spacing 1 mm, FOV 30 cm × 30 cm, matrix size, 240 × 320) were included in each examination. The MRI was examined by two neuroradiologists who were unaware of the patient's identity or response to treatment. The extent of resection was determined by postoperative MRI. Total resection indicated the absence of residual tumor; subtotal resection indicated resection of ≥90% of the tumor.

### Definition of “mosaic sign”

**Figure 1:**
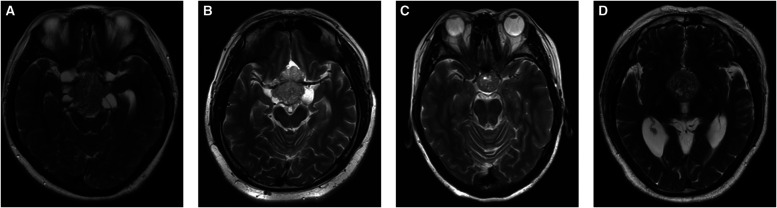
Radiological features of “Mosaic Sign”. (**A–D**) Sagittal T2-WI in 4 patients showed intratumoral hyperintense dots within the tumor.

“Mosaic sign” was defined as a lesion containing small multiple hyperintense dots (ranging in size from 0.5 mm to <2 mm), or cystic changes that are predicted to be a soft-tissue compartment of the adenoma. However, if the tumor presented with a homogenous hypointense signal without a mosaic sign, it predicted the tumor compartment to be fibrous (Figure 1) (14).

### Histopathological examination

All surgical specimens were treated as usual. Tissues were fixed in 10% formalin buffer and embedded in paraffin. 4 µm tissue sections were prepared for immunohistochemical analysis.

The tumors were classified according to the fourth edition of the WHO Classification of Pituitary Tumors (17).

### Statistical analysis

Statistical analyses were performed using SPSS version 21.0. Categorical variables were defined by frequency and percentage rate, and numeric variables with mean ± standard deviation (SD). In triple independent group comparisons, ANOVA tests were used for normally distributed numeric variables, and Kruskal–Wallis tests were used for non-normally distributed data. Categorical variables were compared using a Chi-square test. Statistically significant results were defined with a *P*-value of <0.05.

## Results

### Findings of basic data

A population of 137 patients was enrolled in this cohort, including 63 (46.0%) males and 74 (54.0%) females. The average age of surgery was 55.3 ± 16.9 years. According to the T2-WI manifestation and the classification of Knosp or Hardy (Table 1). However, we found a statistically significant difference in BMI values between the two groups. Patients without the “Mosaic sign” were higher than patients with the “Mosaic sign” (*P* = 0.0280).

**Table 1:** Clinical features, imaging, surgical, and pathological details of 137 patients with PitNETs.

	Patients with the “Mosaic sign” (*n* = 43)	Patients without the “Mosaic sign” (*n* = 94)	*P*
Age ± STD	53.4 ± 16.9	56.6 ± 15.9	0.2749
Gender (male/female)	20/23	43/51	0.9334
BMI (kg/m^2^)	24.9 ± 3.5	26.3 ± 3.3	0.0280*
Primary surgery	30 (69.8%)	66 (70.2%)	0.8864
Knosp			
1	8	13	0.4716
2	13	32	0.6595
3	16	39	0.6353
4	6	10	0.5750
Hardy			
A	11	34	0.2207
B	13	25	0.6590
C	7	20	0.4950
D	10	12	0.1207
E	2	5	0.8691
Tumor types			
Somatotroph tumors (*n*)	6 (14.0%)	26 (27.7%)	0.0785
Lactotroph tumors (*n*)	5 (11.6%)	19 (20.2%)	0.2199
Corticotroph tumors (*n*)	16 (37.2%)	19 (20.2%)	0.0343*
Gonadotroph tumors (*n*)	11 (25.6%)	21 (22.3%)	0.6774
Plurihormonal tumors (*n*)	1 (2.3%)	3 (3.2%)	0.7800
Null cell tumors(*n*)	4 (9.3%)	6 (4.2%)	0.5421
Tumor consistency			<0.0001*
Fibrous (*n*)	0 (0%)	40 (42.6%)	
Soft (*n*)	43 (100%)	54 (57.4%)	
Resection range			0.0288
Total (*n*)	42 (97.6%)	80 (85.1%)	
Subtotal (*n*)	1 (2.4%)	14 (24.9%)	
CSF leakage (*n*)	0 (0%)	1 (1.1%)	0.4972
Hospitalization days	8.5 ± 3.7	7.5 ± 4.0	0.0799

The symbol * represents *p* < 0.05.

### Findings of surgery

**Figure 2:**
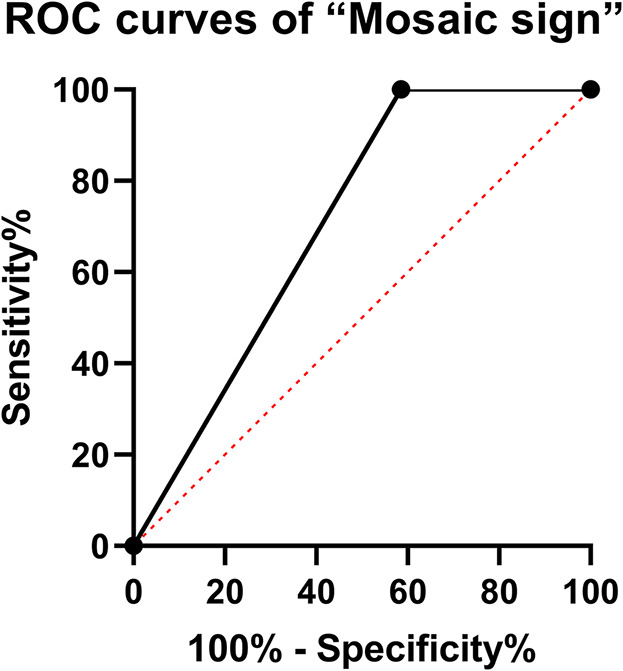
ROC curves of “Mosaic sign”. Sensitivity and specificity of 70.7% and 100%, respectively.

Intraoperatively, all of the 43 (100%) patients had tumors that were classified as soft in the group of patients with the “Mosaic sign”. In addition, 54 (57.4%) patients had tumors that were classified as soft, and 40 (42.6%) patients were found to have fibrous tumors in the other group. The difference was statistically significant between the two groups (*P* < 0.0001). The ROC curve and the area under the ROC curve (AUC) were generated to evaluate the “Mosaic sign” potential use as a predictor of tumor consistency with a sensitivity and specificity of 70.7% and 100%, respectively (Figure 2). The group of “Mosaic sign” gross total resection was achieved in 42 (97.6%) patients, subtotal resection (>90% of tumor) in 1 (2.4%) patient. Among patients without the “Mosaic sign”, 80 cases (85.1%) had a gross total resection, and 14 cases (24.9%) had subtotal resections (estimated resection of >90%). This difference was statistically significant (*P* = 0.0288). (Table 1). In the “Mosaic sign” group, 22 cases had Knosp grade ≥3, and 19 cases had Hardy grade ≥C (This type of tumor is considered invasive (18, 19)). Only subtotal resection was performed in 1 patient because the tumor enveloped the internal carotid artery and the adhesion was tight. By contrast, in the other group, subtotal resection was performed in 14 of 49 PitNETs of Knosp grades 3 and 4 (28.5%), and 11 cases (29.7%)were subtotal resections in PitNETs of Hardy grade ≥C (Table 2).

**Table 2:** Invasion and degree of tumor resection.

	Total Resection (*n*)	Subtotal Resection (*n*)
“Mosaic sign”		
Knosp ≥3	22	0
Hardy ≥C	18	1
CSF leakage (*n*)	0	0
Non-“Mosaic sign”		
Knosp ≥3	35	14
Hardy ≥C	26	11
CSF leakage(*n*)	1	0

### Findings of pathologic analyses

16 cases (37.2%) in the group of “Mosaic sign” were corticotroph tumors, which was higher than that in the non-Mosaic group19 cases (20.2%), which (*P* = 0.0343). There was no statistical difference among other types (Somatotroph adenomas, *P* = 0.0785; Lactotroph tumors, *P* = 0.2199; Gonadotroph tumors, *P* = 0.6774; Plurihormonal tumors, *P* = 0.7800; Null cell tumors, *P* = 0.5421) (Table 1).

### Illustrative cases

**Figure 3:**
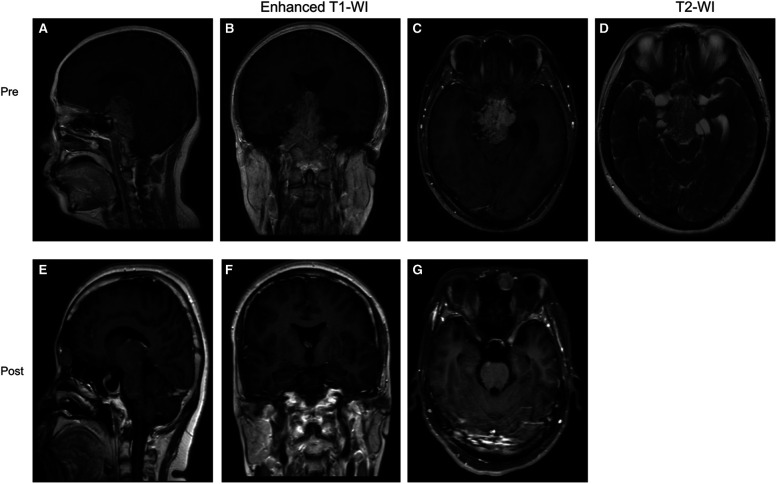
Preoperative and postoperative MRI images of a patient in the “Mosaic sign” group. (**A–C**) Preoperative coronal, axial, and sagittal enhanced MRI, Knosp = 3, Hardy = D; (**D**) Preoperative sagittal T2-WI with “Mosaic Sign”; (**E–G**) The tumor was completely resected on coronal, axial, sagittal enhanced MRI 1 month postoperatively.

Case 1 was a 39-year-old female patient who presented with vision loss. Pre-op. A huge invasive tumor located in the sellar and suprasellar region was found on MRI. The tumor presented a “Mosaic sign” on T2-WI, hence it was predicted to be a soft tumor. So, we chose an EEA to remove the tumors, which were confirmed to be soft (Supplementary Figure S1). Post-op. No complications such as CSF leakage, intra-cranial infection, or hypopituitarism occurred after surgery (Figure 3). The immunohistochemical result proved to be a silent ACTHoma (Supplementary Figure S2). (GH (−), PRL (−), LH (−), TSH (−), FSH (−), ACTH (+), Ki-67 (1–3%), PIT-1 (−), SF-1 (+), T-PIT (+)).

**Figure 4:**
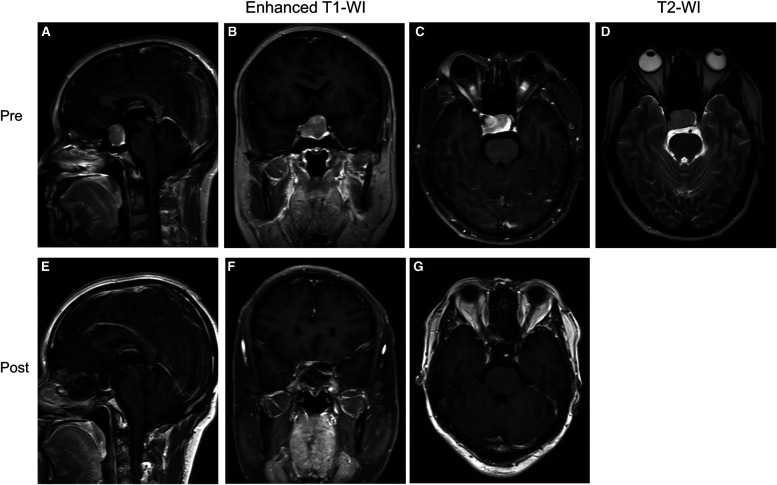
Preoperative and postoperative MRI images of a patient in the group without “Mosaic sign”. (**A–C**) Preoperative coronal, axial, and sagittal enhanced MRI, Knosp = 2, Hardy = B; (**D**) Preoperative sagittal T2-WI without “Mosaic Sign”; (**E–G**) The tumor was completely resected on coronal, axial, sagittal enhanced MRI 1 month postoperatively.

Case 2 was a 47-year-old male patient who presented with vision loss and headache. Pre-op. The neoplasm showed uniform hyposensitivity on T2-WI and was therefore predicted to be a fibrous tumor. Because the tumor growth was relatively regular, we chose EEA to remove the tumor, which was proved to be fibrous (Supplementary Figure S1). Post-op. No complications such as CSF leakage, intra-cranial infection, or hypopituitarism occurred after surgery (Figure 4). The immunohistochemical result proved to be a nonfunction tumor (Supplementary Figure S2). (GH (−), PRL (−), LH (−), TSH (−), FSH (+), ACTH (−), Ki-67 (2–4%), PIT-1 (−), SF-1 (−), T-PIT (−)).

## Discussion

### Prediction of tumor consistency

The preoperative consistency prediction of PITnet is controversial, and different imaging has its unique value. For example, Wan et al. made consistent predictions based on a radiomic model of multi-parameter magnetic resonance imaging (mpMRI), while Cohen-Cohen et al. argued that MRE was a reliable tool compared to other sequences (20, 21). As far as we know, currently, studies on preoperative prediction of tumor consistency have focused on imaging findings on T2-WI (11, 22). Although there are controversies, it seems that T2-WI strongly indicates tumor consistency (23). Some studies have shown that a low signal on T2-weighted images corresponds to fibrotic tumors. Some people believe that a signal of equal intensity is more likely to predict fibrotic tumors. Finally, some researchers believe that there is no significant relationship between tumor consistency and MRI (10, 16, 24). One study showed that tumors may be fibrous if showing low signal strength and homogeneous enhancement on T2-WI (10). Smith et al. suggested that tumor-to-cerebellar peduncle ratios could predict tumor consistency. Ratios >1.8 have a high predictive value for soft consistency tumors; ratios <1.5 have a high predictive value for firm consistency tumors (16). However, some studies have concluded that relative signal strength values do not correspond to tumor consistency (15, 25). The main reason may be that the factors influencing T2-WI signal strength are independent of the histological results (26). Based on existing reports, we found that PitNETs with mixed signals of high and low intensity present in T2-WI which we called “Mosaic sign” usually indicates that the tumor is soft and easy to remove. The judgment of signal level is only the comparison of the signal inside the tumor and has nothing to do with the factors outside the tumor, avoiding the error brought by comparing the gray matter or other tissues with the tumor tissue. In this study, all the tumors with “Mosaic sign” had a soft consistency. The causes of such imaging are complex, uneven tumor cell density, or uneven free water, fiber, and collagen content in different parts of the tumor, or the presence of multimicrocystic. There is evidence that tumors with more collagen show lower signal intensity on T2-weighted images (10). From our point of view, when tumor components are mixed, that is, collagen and free water are mixed and dispersed between tumors, there will be “Mosaic” markers, which may be related to tumor growth rate and growth mode, which needs to be further explored. Furthermore, there is a special case, that is, scattered small cystic changes within the tumor.

### “Mosaic sign” for surgical selection

At present, the surgical approach for the giant tumor is still controversial. For larger lesions, the consistency of the tumor may be a factor in determining the need for craniotomy (27). If the tumor consistency is soft, endoscopic transnasal surgery can achieve satisfactory results (28). Meanwhile, the consistency of PitNET is an important intraoperative characteristic that may dictate operative dissection techniques and/or instruments used for tumor removal during endoscopic endonasal approaches (6, 29). Furthermore, preoperative determination of tumor consistency can minimize the chance of postoperative complications and residual tumors (17). Tumors with soft consistency are easy to be sucked out/curetted intraoperatively, and the effect of resection is better. Secondly, if there is a suprasellar extension of the tumor, after intraoperative resection of the lower part of the tumor, the saddle of the tumor is easy to descend, and the complete resection of the tumor can be completed at one time, avoiding the secondary operation or even craniotomy. Finally, the soft tumors are more likely to separate from the surrounding tissue and proper intraoperative use of aspirators can help the surgeon remove the tumor adequately, causing less damage to the surrounding tissue. Fibrous tumors are more difficult to remove with a transsphenoidal approach. Internal debulking can be difficult even with the use of an ultrasound aspirator, and the fragmentation cannot be easily accomplished without adequate mobilization (28). Moreover, it is difficult to peel off such tumors that adhere to the surrounding tissues with ordinary instruments, which is easy to cause damage to the surrounding tissues. At this time, transcranial approaches or combined transcranial and endoscopic approaches are necessary (30). Therefore, preoperative assessment and prediction of tumor consistency are particularly important. The improved preoperative prediction may better guide patients on risks and benefits. In our study cohort, it is not difficult to find that for giant tumors, transnasal endoscopic surgery in the “Mosaic sign” group of cases is more likely to achieve satisfactory results, and there are no significant complications. One of the main reasons is that tumors with the “Mosaic sign” tend to be softer and easier to remove during surgery. There is no denying that the surgeon's skill and experience can also affect the outcome. In conclusion, endoscopic transsphenoidal surgery can be selected even if the pituitary tumor is large if there is a “Mosaic” sign on T2-weighted images before surgery, and better surgical results can be achieved.

### “Mosaic sign” and tumor types

Notably, the pathological type of the tumor was correlated with the “Mosaic sign”, that is, corticotroph tumors (Including SCAs) were more prone to the “Mosaic sign”, suggesting soft tumor consistency, consistent with what has been reported in the literature (31). Microcyst patterns on T2-WI have been considered radiological features of SCAs in several studies (32, 33). Laure et al. found multiple microcysts in most SCAs and pseudopapillary artefactual dehiscences and perivascular pseudorosettes in SCAs on pathological examination. They considered that a dissociated tissue with pseudopapillary dehiscences could explain the small hyperintense foci in T2-WI (34). The radiographic appearance of this microcyst coincides with our definition of a “Mosaic sign”. However, the mechanism needs further research.

### Limitation

Our study has several limitations. Firstly, this study was a retrospective analysis with a relatively small number of patients. Secondly, the quantitative description of the “Mosaic sign” was not included in our study. In future work, we can quantitatively describe such imaging findings by reviewing more case data. Third and finally, we did not quantitatively analyze the degree of fibrosis in histopathological specimens, and we will verify this in the future.

## Conclusions

The “Mosaic sign” on T2-WI in patients with PitNET may indicate a soft tumor texture, and a satisfactory resection can be achieved by endoscopic transnasal surgery, even for large, aggressive tumors. But further large-scale studies are needed to confirm and improve this approach.

## Data Availability

Some or all datasets generated during and analyzed during the current study are not publicly available but are available from the corresponding author on reasonable request.

## Supplementary material

Supplementary Figure 1

Intraoperative conditions. (A-B) The texture of the tumor is soft; (C) The texture of the tumor is fibrous.

Supplementary Figure 2

Hematoxylin-eosin staining. (A) Case 1; (B) Case 2.
