# Supplementary figures and images for: Application of “mosiac sign” on T2-WI in predicting the consistency of pituitary neuroendocrine tumors
Source: Front Surg. 2022 Jul 26;9:922626. doi: 10.3389/fsurg.2022.922626 (PMC9360528; doi:10.3389/fsurg.2022.922626)

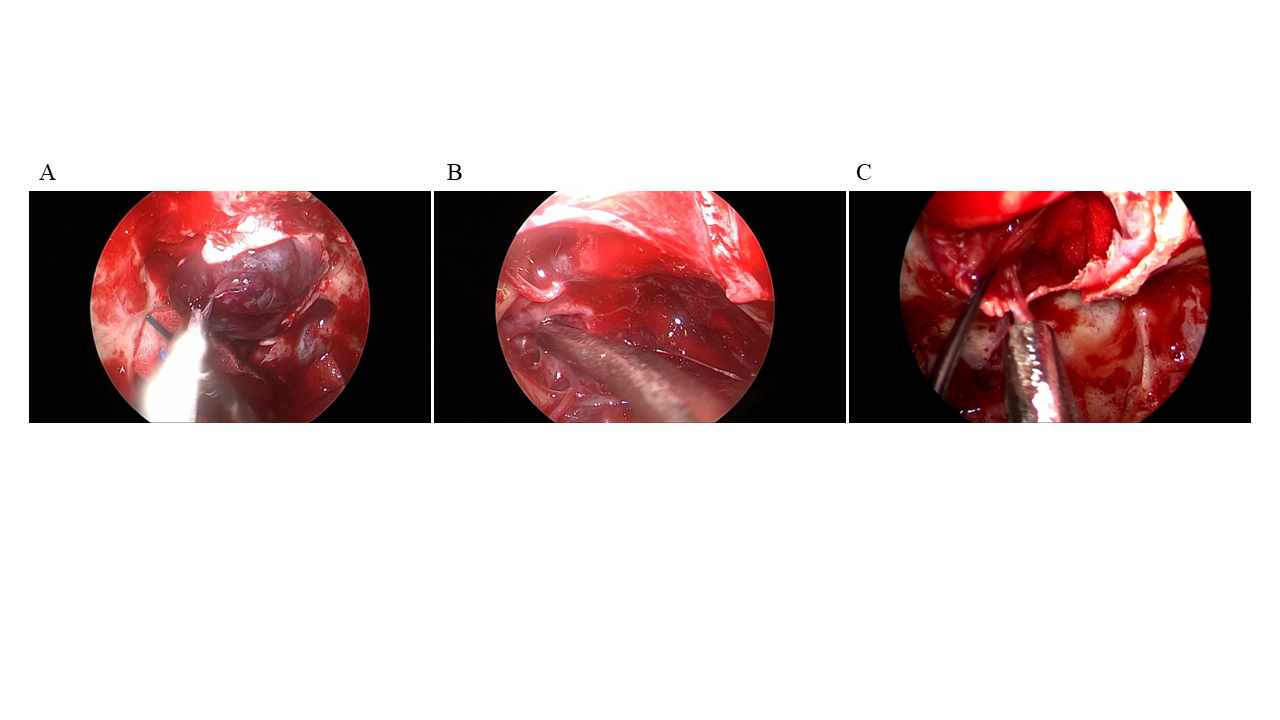

Supplement: Supplementary Figure 1 [file Image_1_v1.tif]

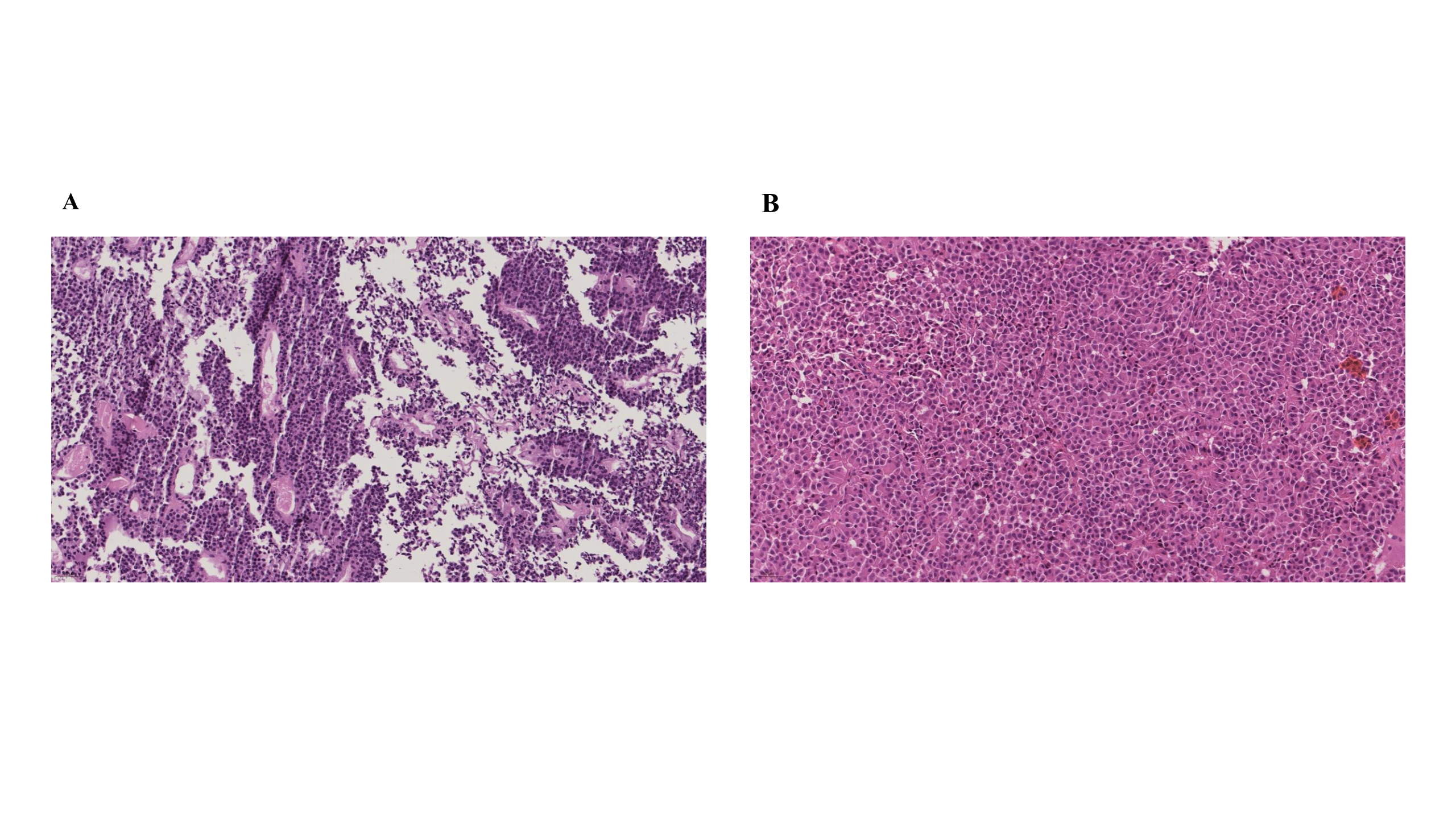

Supplement: Supplementary Figure 2 [file Image_2_v1.tif]
